# BAYONET trial: staged combination with encorafenib, binimetinib, plus cetuximab following encorafenib plus cetuximab for *BRAF* V600E-mutant metastatic colorectal cancer

**DOI:** 10.1016/j.esmogo.2024.100066

**Published:** 2024-06-03

**Authors:** Y. Matsubara, H. Bando, D. Kotani, Y. Kagawa, K. Harada, H. Osumi, N. Izawa, T. Kawakami, S. Boku, T. Matsumoto, M. Wakabayashi, T. Yoshino

**Affiliations:** 1 Department of Clinical Oncology, Aichi Cancer Center Hospital, Aichi; 2 Department of Gastroenterology and Gastrointestinal Oncology, National Cancer Center Hospital East, Kashiwa; 3 Translational Research Support Section, National Cancer Center Hospital East, Kashiwa; 4 Department of Gastroenterological Surgery, Osaka General Medical Center, Osaka; 5 Department of Gastroenterology and Hepatology, Hokkaido University Hospital, Sapporo; 6 Department of Gastroenterological Chemotherapy, Cancer Institute Hospital of Japanese Foundation for Cancer Research, Tokyo; 7 Department of Clinical Oncology, St. Marianna University School of Medicine, Kawasaki; 8 Division of Gastrointestinal Oncology, Shizuoka Cancer Center, Shizuoka; 9 Cancer Treatment Center, Kansai Medical University Hospital, Osaka; 10 Medical Oncology, Ichinomiya Nishi Hospital, Aichi; 11 Division for the Promotion of Drug and Diagnostic Development, National Cancer Center Hospital East, Kashiwa, Japan

**Keywords:** *BRAF* V600E, metastatic colorectal cancer, encorafenib, binimetinib, cetuximab

## Abstract

**Background:**

While the triplet combination of encorafenib (ENCO), binimetinib (BINI), plus cetuximab (CET) yielded a higher response rate compared with the doublet combination of ENCO plus CET, no significant survival benefits of the triplet combination were observed in patients with *BRAF* V600E-mutant metastatic colorectal cancer (mCRC), according to the BEACON CRC study. Although ENCO plus CET is the standard second-line therapy, poor prognoses are expected after disease progression.

**Trial design:**

BAYONET is a single-arm multicenter phase II trial designed to evaluate the efficacy and safety of staged combination with ENCO, BINI, plus CET for patients with *BRAF* V600E-mutant mCRC refractory to ENCO plus CET. The main inclusion criteria are as follows: *RAS* wild-type/*BRAF* V600E-mutant mCRC; <4 weeks from the last administration of previous ENCO or CET; no administration of other systemic therapy after refractoriness to ENCO plus CET; and complete response, partial response, or ≥4 months of stable disease in the previous ENCO plus CET. The primary endpoint of this trial is the 12-week progression-free survival rate. As a translational analysis, circulating tumor DNA for next-generation sequencing using Guardant360 is collected at two time points (before and after study treatment) to investigate potential mechanisms of resistance.

## Highlights

• *BRAF*-V600E mutant mCRC exhibits poor outcomes after treatment with ENCO plus CET.
• BAYONET is a single-arm, multicenter phase II trial designed to evaluate ENCO, BINI, plus CET for *BRAF* V600E-mutant mCRC refractory to ENCO plus CET.
• The primary endpoint is the 12-week progression-free survival rate.

## Graphical abstract

**Table:** Specifications table

Subject area	Medicine and Dentistry
**More specific subject area**	*BRAF* V600E-mutant colorectal cancer
**Name of your trial in progress**	BAYONET trial
**Reagents/tools**	Eligible patients who participated in this study received the combination treatment of encorafenib (ENCO; 300 mg once a day), binimetinib (BINI; 45 mg twice a day), plus cetuximab (CET; 400 mg/m^2^ initial dose and then 250 mg/m^2^ once a week) in a 28-day cycle as a study treatment until disease progression, death, patient’s withdrawal, or investigator’s decision (whichever comes first).
**Trial design**	BAYONET is a single-arm multicenter phase II trial designed to evaluate the efficacy and safety of staged combination with ENCO, BINI, plus CET for patients with *BRAF* V600E-mutant mCRC refractory to ENCO plus CET. The main inclusion criteria are as follows: *RAS* wild-type/*BRAF* V600E-mutant mCRC; <4 weeks from the last administration of previous ENCO or CET; no administration of other systemic therapy after refractoriness to ENCO plus CET; and complete response, partial response, or ≥4 months of stable disease observed in the previous ENCO plus CET. Included patients receive ENCO (300 mg once a day), BINI (45 mg twice a day), plus CET (400 mg/m^2^ initial dose and then 250 mg/m^2^ once a week) in a 28-day cycle as a study treatment. The planned sample size is 30. The primary endpoint of this trial is the 12-week progression-free survival (PFS) rate. The secondary endpoints are PFS, overall survival, objective response rate, disease control rate, time to treatment failure, and the incidence of adverse events. As a translational analysis, circulating tumor DNA for next-generation sequencing using Guardant360 is collected at two time points (before and after study treatment) to investigate potential mechanisms of resistance.
**Trial registration**	jRCTs031210510
**Ethics**	The current study has been approved by the Certified Institutional Review Board of National Cancer Center Hospital East (K2021006), and permission for conducting the study has been obtained from the management of all participating facilities. All patients are required to sign written informed consent. All experiments were carried out in accordance with the Declaration of Helsinki.
**Value of the trial in progress**	• A staged combination of ENCO, BINI, plus CET may overcome resistance to ENCO plus CET and has the potential to be an effective treatment strategy for patients with *BRAF* V600E-mutant mCRC. • A prospective, translational analysis using circulating tumor DNA is expected to reveal potential mechanisms underlying resistance to BRAF inhibitor plus anti-epidermal growth factor receptor (EGFR) antibody, contributing to the development of future promising treatments.

## Description of protocol

### Background

While the triplet combination of encorafenib (ENCO), binimetinib (BINI), plus cetuximab (CET) yielded a higher response rate compared with the doublet combination of ENCO plus CET, no significant survival benefits of the triplet combination were observed in patients with *BRAF* V600E-mutant metastatic colorectal cancer (mCRC), according to the BEACON CRC study.^1^^,^^2^ Although ENCO plus CET is the standard second-line therapy in the United States and European Union, poor prognoses are expected after disease progression. Several mitogen-activated protein kinase (MAPK) pathway mutations, including *RAS*, *RAF*, and *MEK,* have been reported and are possible mechanisms of resistance to BRAF plus epidermal growth factor receptor (EGFR) blockage, suggesting that additional blockade of MAPK signaling may be an effective strategy for ENCO plus CET refractory mCRC.^3^, ^4^, ^5^, ^6^, ^7^

## Methods/design

### Study design and treatment

**Figure 1:**
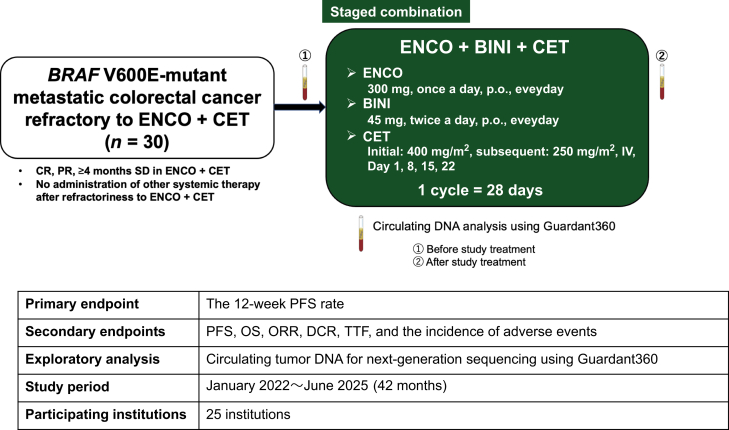
**Phase II single-arm, multicenter study**. BINI, binimetinib; CET, cetuximab; CR, complete response; DCR, disease control rate; ENCO, encorafenib; IV, intravenous; ORR, objective response rate; OS, overall survival; PFS, progression-free survival; p.o., per os (by mouth); PR, partial response; SD, stable disease; TTF, time to treatment failure.

BAYONET is a single-arm, multicenter phase II trial designed to evaluate the efficacy and safety of the staged combination with ENCO, BINI, plus CET for patients with *BRAF* V600E-mutant mCRC who were refractory to ENCO plus CET (Figure 1). Table 1 shows the inclusion and exclusion criteria. The main inclusion criteria are as follows: histologically diagnosed as metastatic colorectal adenocarcinoma (not containing appendix and anal canal cancer); *RAS* wild-type/*BRAF* V600E-mutant mCRC; age ≥20 years; Eastern Cooperative Oncology Group Performance Status of 0 or 1; observed disease progression <4 weeks from the last administration of ENCO or <4 weeks from the last administration of ENCO or CET; no administration of other systemic therapy after failure of ENCO plus CET; complete response (CR), partial response (PR), or ≥4 months of stable disease (SD) observed during previous treatment with ENCO plus CET; no prior administration of MEK inhibitors; expected to be tolerable without an initial dose reduction of ENCO, BINI, plus CET; preserved the main organ function; availability to take oral medication; life expectancy longer than 12 weeks; and be registered or has a plan to participate in the GOZILA study (UMIN000029315). Patients who have Gilbert’s syndrome, UGT1A1∗6/∗6, UGT1A1∗28/∗28, or UGT1A1∗6/∗28∗ were excluded because they may affect the drug metabolism of BINI, whose main metabolizing enzyme is UGT1A1. Other exclusion criteria are as follows: serious complications; receiving prior surgery or antineoplastic drug described in the study protocol; grade 2 or higher adverse events in prior treatment and have not resolved, excluding anemia, hair loss, skin pigmentation, peripheral neuropathy due to oxaliplatin, and hypertension or proteinuria due to angiogenesis inhibitors; a history of a severe allergy to ENCO or CET; histories of severe lung disease; pregnancy, lactating, or have no intention of contraception; unsuitable for the study due to complicated disorders affecting the assessment of toxicity according to the investigator’s judgment.

**Table 1:** Patient inclusion and exclusion criteria

Inclusion criteria
• Patients histologically diagnosed as having metastatic colorectal adenocarcinoma (not containing appendix and anal canal cancer). • Patients with *RAS* (*KRAS*/*NRAS*) wild-type and *BRAF* V600E-mutant metastatic colorectal cancer diagnosed using tissue specimens. • Patients whose best response in the previous encorafenib plus cetuximab trial was complete response, partial response, or ≥4 months of stable disease. • Patients who had disease progression within 4 weeks from the last administration of encorafenib. • Patients who received encorafenib and cetuximab within 4 weeks. • Patients who did not receive any other systemic therapy after refractoriness to encorafenib and cetuximab. • Patients who had no prior administration of MEK inhibitors. • Patients who were registered or planned to participate in the GOZILA study (UMIN000029315). • Patients with Eastern Cooperative Oncology Group Performance Status of 0 or 1 • Patients ≥20 years old at the time point of providing informed consent. • Patients who are able to take oral medication. • Patients who are expected to be tolerable without an initial dose reduction of encorafenib, binimetinib, and cetuximab. • Patients with the following organ function within 14 days of registration. – Neutrophil count ≥1500/mm^3^ – Platelet count ≥75 000 mm^3^ – Hemoglobin ≥9.0 g/dl – Total bilirubin ≥2.0 mg/dl – Aspartate aminotransferase, alanine aminotransferase <100 IU/l – Serum creatinine <1.5 mg/dl or calculated creatinine clearance of ≥50 ml/min, as evaluated using the Cockcroft–Gault method • Patients with life expectancy >3 months. • Patients who sign written informed consent.
Exclusion criteria
• Patients with the following serious complications: – Active multiple primary malignancies. – Brain metastasis or meningeal dissemination that is difficult to control. – Active infection. – Ascites, pleural, or pericardial effusion requiring continuous drainage. – Poorly controlled diabetes mellitus and hypertension. – Acute coronary syndrome, severe/unstable angina, or symptomatic congestive heart failure assessed as New York Heart Association Class III or IV within 6 months. – A history or risk of retinal vein occlusion. – Psychosis or psychiatric symptoms that make patients difficult to participate in clinical research. • Patients who underwent the following treatment or intervention: – Major surgery within 4 weeks. – Stoma construction within 2 weeks. – Any antitumor drugs except encorafenib, cetuximab, fluorouracil, and hormone preparations. • Patients whose adverse events were grade ≥2 in prior treatment and have not resolved, excluding anemia, hair loss, skin pigmentation, peripheral neuropathy due to oxaliplatin, and hypertension or proteinuria due to angiogenesis inhibitors. • Patients with a history of a severe allergy to encorafenib or cetuximab. • Patients with histories of severe lung disease. • Patients who are pregnant, lactating, or have no intention of contraception. • Patients with Gilbert’s syndrome, UGT1A1∗6/∗6, UGT1A1∗28/∗28, or UGT1A1∗6/∗28∗. • Patients unsuitable for the study due to complicated disorders affecting the assessment of toxicity according to the investigator’s judgment.

Eligible patients who participated in this study received the combination treatment of ENCO (300 mg once a day), BINI (45 mg twice a day), plus CET (400 mg/m^2^ initial dose and then 250 mg/m^2^ once a week) in a 28-day cycle as a study treatment until disease progression, death, patient’s withdrawal, or investigator’s decision (whichever comes first). The BAYONET trial was approved by the Certified Review Board of the National Cancer Center Hospital East (K2021006) and registered in the Japan Registry of Clinical Trials (jRCT; jRCTs031210510, 1 January 2022). Enrollment began in January 2022 at 25 facilities in Japan, and it is expected to be completed in June 2024. The principal investigator and the study coordinator immediately respond to inquiries about participation in the BAYONET trial using phone or email.

### Endpoints

The primary endpoint is the 12-week progression-free survival (PFS) rate. The secondary endpoints are PFS, overall survival (OS), objective response rate (ORR), disease control rate (DCR), time to treatment failure (TTF), and incidence of adverse events.

The 12-week PFS is defined as the proportion of the patients whose best response is CR, PR, or SD at the 12-week time point, as measured from the date of registration. The best responses of each patient will be evaluated based on the RECIST, version 1.1. CT scans will be conducted every 6 weeks. We define OS as the time from registration to death and PFS as the time from registration to disease progression or death, whichever occurs earlier. In this study, TTF is the period from registration to discontinuation of protocol treatment. The ORR and DCR are defined as the proportion of treated patients who exhibit CR and PR or CR, PR, and SD, respectively. Adverse events will be evaluated according to the Common Terminology Criteria for Adverse Events (CTCAE), version 5.0.

### Safety assessment

The research office will periodically monitor whether the actual adverse events are within the expected range; if serious or unexpected adverse events occur, the efficacy and safety of treatment will be evaluated in accordance with the relevant regulations. A system for reporting severe adverse events (SAEs) to the Efficacy and Safety Evaluation Committee has been established, and necessary measures will be taken. SAE is defined as the following events related to the study treatment: death, adverse events that may lead to death or require hospitalization at a medical institution for treatment or an extension of the period of hospitalization, disability, diseases that may lead to disability, or congenital diseases or abnormalities in subsequent generations. Death and adverse events that may lead to death are planned to be addressed to the principal investigator within 72 h. Other SAEs are reported within 10 days. Upon receiving the report, the principal investigator will judge the urgency, importance and degree of impact of the content of the report, among others, and take measures or make urgent contact, if necessary, such as suspending registration or disseminating information to participating institutions. The dose of each drug should be modified according to the dose modification criteria in the study protocol. The observation period for adverse events that occur during the study will start from the date of study treatment up to 30 days after the final treatment date or before the start of post-treatment, whichever comes first.

### Sample size and statistical analysis

According to the control arm in the BEACON CRC study, the threshold of the primary endpoint was set at 20%.^1^^,^^2^ Considering that *BRAF* V600E-mutant mCRC has a poor prognosis, the expected value was set at 40%. According to these statistical settings, the required sample size was calculated to be 30, using a method based on a binomial distribution with a one-sided alpha of 10% and a power of 80%.

### Translational analysis

Eligible patients will also participate in the GOZILA study (UMIN000029315), an observational circulating tumor DNA-based screening study in Japan. Circulating tumor DNA is collected at two time points (before and after study treatment) for next-generation sequencing using Guardant360 to investigate the potential resistance mechanisms. Guardant360 is a liquid biopsy test that can detect 74 cancer-associated gene alterations by extracting circulating tumor DNA from blood samples using proprietary digital sequencing technology developed by Guardant Health.^8^

## Discussion

The BAYONET trial is the first phase II trial that evaluates the staged combination with a triplet combination of BRAF inhibitor, MEK inhibitor, plus anti-EGFR antibody for patients with *BRAF* V600E-mutant mCRC who are refractory to the doublet combination of BRAF inhibitor plus anti-EGFR antibody. Additional blockade of the MAPK pathway should overcome the resistance to BRAF inhibitor plus anti-EGFR antibody. In addition, a prospective, translational analysis is expected to reveal potential mechanisms underlying resistance to BRAF inhibitor plus anti-EGFR antibody, contributing to the development of future promising treatments. Patients with primary resistance to ENCO plus CET are excluded because this study aims to overcome acquired resistance to BRAF inhibitor plus anti-EGFR antibody. The ORR is traditionally used as a key endpoint for phase II single-arm trials. However, in this study, we chose not to use ORR as the main measure. This decision was driven by our focus on evaluating the additional benefits of an MEK inhibitor with ENCO and CET following treatment with ENCO plus CET. While we anticipated limited tumor reduction, our primary expectation was prolonged tumor stability. Because of these considerations, we selected the 12-week PFS rate as the primary endpoint. The MEK inhibitor is not approved for *BRAF* V600E-mutant mCRC in countries other than Japan as the triplet combination of ENCO, BINI, plus CET and the doublet combination of ENCO plus CET showed similar OS and PFS in the BEACON CRC study.^1^^,^^2^ The BAYONET trial has the potential to demonstrate the role of MEK inhibitors in patients with *BRAF*-V600E mCRC who are refractory to ENCO plus CET. As a global phase III trial, the BREAKWATER trial (NCT04607421), which evaluates the combination of ENCO plus CET with or without chemotherapy as a first-line treatment for patients with *BRAF* V600E-mutant mCRC, is ongoing. If ENCO, CET, plus chemotherapy and/or ENCO plus CET replaces the standard first-line treatment for mCRC, the results of the BAYONET trial will be indispensable considering second-line treatment after refractoriness to BRAF inhibitor plus anti-EGFR antibody.

A staged combination with ENCO, BINI, plus CET should overcome treatment resistance to ENCO plus CET and can be an effective treatment strategy for patients with *BRAF* V600E-mutant mCRC.
